# Evaluation of Commercially Available High-Throughput SARS-CoV-2 Serologic Assays for Serosurveillance and Related Applications

**DOI:** 10.3201/eid2803.211885

**Published:** 2022-03

**Authors:** Mars Stone, Eduard Grebe, Hasan Sulaeman, Clara Di Germanio, Honey Dave, Kathleen Kelly, Brad J. Biggerstaff, Bridgit O. Crews, Nam Tran, Keith R. Jerome, Thomas N. Denny, Boris Hogema, Mark Destree, Jefferson M. Jones, Natalie Thornburg, Graham Simmons, Mel Krajden, Steve Kleinman, Larry J. Dumont, Michael P. Busch

**Affiliations:** Vitalant Research Institute, San Francisco, California, USA (M. Stone, E. Grebe, H. Sulaeman, C. Di Germanio, H. Dave, K. Kelly, G. Simmons, L.J. Dumont, M.P. Busch);; University of California–San Francisco, San Francisco (M. Stone, E. Grebe, G. Simmons, M.P. Busch);; South African Centre for Epidemiological Modelling and Analysis, Stellenbosch University, Stellenbosch, South Africa (E. Grebe);; Centers for Disease Control and Prevention, Fort Collins, Colorado, USA (B.J. Biggerstaff);; University of California Irvine Medical Center, Orange, California, USA (B.O. Crews);; University of California–Davis, Davis, California, USA (N. Tran);; Fred Hutchinson Cancer Research Center, University of Washington, Seattle, Washington, USA (K.R. Jerome);; Duke Human Vaccine Institute, Duke University, Durham, North Carolina, USA (T.N. Denny);; Sanquin Research, Amsterdam, the Netherlands (B. Hogema); BloodWorks NorthWest, Seattle (M. Destree);; Centers for Disease Control and Prevention, Atlanta, Georgia, USA (J.M. Jones, N. Thornburg);; British Columbia Centre for Disease Control, Vancouver, British Columbia, Canada (M. Krajden);; University of British Columbia, Vancouver (S. Kleinman);; University of Colorado School of Medicine, Denver, Colorado, USA (L.J. Dumont)

**Keywords:** COVID-19, coronavirus disease, SARS-CoV-2, severe acute respiratory syndrome coronavirus 2, viruses, respiratory infections, zoonoses, vaccine-preventable diseases, serologic assays

## Abstract

Severe acute respiratory syndrome coronavirus 2 (SARS-CoV-2) serosurveys can estimate cumulative incidence for monitoring epidemics, requiring assessment of serologic assays to inform testing algorithm development and interpretation of results. We conducted a multilaboratory evaluation of 21 commercial high-throughput SARS-CoV-2 serologic assays using blinded panels of 1,000 highly characterized specimens. Assays demonstrated a range of sensitivities (96%–63%), specificities (99%–96%), and precision (intraclass correlation coefficient 0.55–0.99). Durability of antibody detection was dependent on antigen and immunoglobulin targets; antispike and total Ig assays demonstrated more stable longitudinal reactivity than antinucleocapsid and IgG assays. Assays with high sensitivity, specificity, and durable antibody detection are ideal for serosurveillance, but assays demonstrating waning reactivity are appropriate for other applications, including correlation with neutralizing activity and detection of anamnestic boosting by reinfections. Assay performance must be evaluated in context of intended use, particularly in the context of widespread vaccination and circulation of SARS-CoV-2 variants.

Serosurveillance for severe acute respiratory syndrome coronavirus 2 (SARS-CoV-2) infection is critical to monitor the course of the evolving pandemic and local outbreaks and can provide data on infection-fatality ratios, vaccine coverage, the effect of mitigation measures, and levels of population immunity. Serosurveillance should be conducted with representative population sampling using well-characterized serologic assays selected on the basis of their performance characteristics and optimized algorithms. Using assays and algorithms that detect mild or asymptomatic infections is critical for accurately estimating cumulative incidence, and case-to-infection and death-to-infection ratios.

More than 85 SARS-CoV-2 antibody assays had received US Food and Drug Administration Emergency Use Authorization as of August 19, 2021, ranging from point-of-care tests to fully automated high-throughput platforms (*1*). These assays target different immunoglobulins (total or selective IgG, IgM, or IgA) against viral antigens (full-length spike protein [S1/S2], subunit 1 [S1], subunit 2 [S2] of spike, the receptor binding domain [RBD] of spike, or the nucleocapsid protein [NC]) (*1*). Limited head-to-head evaluation data are available for high-throughput SARS-CoV-2 serologic assays, and few large-scale studies have focused on performance for serosurveillance applications. Comprehensive characterization of assay performance must include sensitivity, specificity, and durability of antibody detection over time since infection.

To provide a comprehensive overview and direct comparison of assay characteristics and performance to inform assay selection and results interpretation for serosurveillance, we conducted a multilaboratory comparative assessment of 21 high-throughput, commercially available SARS-CoV-2 serologic assays by using blinded panels of 1,000 highly characterized specimens, including longitudinal and cross sectional coronavirus disease (COVID-19) convalescent plasma (CCP) and prepandemic control plasma specimens. We distributed panels to experienced testing laboratories that were deemed to be proficient by the manufacturers and selected assays to represent multiple formats and antigen targets. Data from this study can inform assay selection and development of testing algorithms to meet the optimal performance characteristics for primary screening and supplemental testing in US and global serosurveillance studies. The study also provides performance data relevant to other serologic testing contexts that will enable clinicians, public health organizations, laboratorians, and emergency response planners to develop optimal algorithms for infection detection and confirmation, including vaccine breakthrough and recurrent infections and correlations with neutralizing activity.

## Methods

### Assay Selection, Panel Development, and Testing

The study included assays from major manufacturers that were commercially available, were high-throughput, had received or were expected to receive Emergency Use Authorization, and were widely used for serosurveillance (*2*–*8*; S. Takahashi et al., unpub. data, https://doi.org/10.1101/2021.09.09.21263139) or other purposes. In some cases, we included additional assays from a manufacturer not necessarily ideal for serosurveillance applications but still informative to related applications. Key assay characteristics included format and configuration, antigen composition, and immunoglobulin target (Table 1). We distributed uniquely blinded panels consisting of 1,000 identical specimens to experienced testing laboratories to determine performance characteristics.

**Table 1:** Key characteristics of assays evaluated in study of commercially available high-throughput SARS-CoV-2 assays for serosurveillance*

Manufacturer	Assay†	Ig target	Antigen	Assay format	Reported units	Testing laboratory
Ortho	VITROS Immunodiagnostic Products Anti-SARS-CoV-2 Total Ig	Total Ig	S1	Double-antigen sandwich CLIA	S/CO	Vitalant Research Institute
	VITROS Immunodiagnostic Products Anti-SARS-CoV-2 IgG	IgG	S1	Double-antigen sandwich CLIA	S/CO	CTS
EUROIMMUN	Anti-SARS-CoV-2 NCP ELISA	IgG	N	Indirect IgG EIA	S/CO	Advent Health
	Anti-SARS-CoV-2 ELISA	IgG	S1	Antigen sandwich ELISA	S/CO	
	Anti-SARS-CoV-2 QuantiVac ELISA	IgG	S1	Antigen sandwich ELISA	RU/mL	
	Anti-SARS-CoV-2 ELISA	IgA	S1	Antigen sandwich ELISA	S/CO	
Roche	Elecsys Anti-SARS-CoV-2 N on cobas	Total Ig	N	Double-antigen sandwich CLIA	COI	University of California–Davis
	Elecsys Anti-SARS-CoV-2 S on cobas	Total Ig	S1/S2/RBD	Double-antigen sandwich CLIA	U/mL	
DiaSorin	LIAISON 28 SARS-CoV-2 TrimericS IgG	IgG	TrimericS	IgG magnetic particle CLIA	AU/mL	British Columbia Centers for Disease Control and Prevention
Siemens	ADVIA Centaur SARS-CoV-2 Total Ig	Total Ig	S1/RBD	Ag sandwich CLIA	S/CO	
	ADVIA Centaur SARS-CoV-2 IgG	IgG	S1/RBD	Ag sandwich CLIA	Index	
Abbott	SARS-CoV-2 IgG N on ARCHITECT	IgG	N	CMIA	AU/mL	Duke Human Vaccine institute
	SARS-CoV-2 IgG S1 on ARCHITECT	IgG	S1	CMIA	S/CO	
	SARS-CoV-2 IgG N on Alinity	IgG	N	CMIA	S/CO	Fred Hutchinson Cancer Research Center
	SARS-CoV-2 IgG S1 on Alinity	IgG	S1	CMIA	AU/mL	
Bio-Rad	Platelia SARS-CoV-2 Total Ab (Evolis)	Total Ig	N	One-step antigen capture	S/CO	BloodWorks NorthWest
	BioPlex 2200 SARS-CoV-2 IgG Panel	IgG	RBD, S1, S2, N	Multiplexed microbeads two-step assay	S/CO	
Quotient	MosaiQ COVID-19 Antibody Microarray	IgM/IgG	S1/S2	Array	Qualitative only	
Diazyme	DZ-Lite SARS CoV-2	IgG	N & S1/S2	IgG microbead CLIA	S/CO	
Beckman Coulter	Access SARS-CoV-2 IgG	IgG	S1 RBD	IgG 2-step paramagnetic particle CLIA	S/CO	University of California–Irvine
Wantai	SARS-CoV-2 Total Ig	Total Ig	S1 RBD	Total Ig sandwich ELISA	S/CO	Sanquin

*Ab, antibody; Ag, antigen; AU, arbitrary units; CMIA, chemiluminescent microparticle immunoassay; CLIA, chemiluminescent immunoassay;.COVID-19, coronavirus disease; EIA, enzyme immunoassay; Ig, immunoglobulin; N, nucleocapsid; RBD, receptor binding domain; RU, relative units; S, spike protein; SARS-CoV-2, severe acute respiratory syndrome coronavirus 2; S/CO, signal to cutoff ratio; †Current US regulatory status available at https://www.fda.gov/medical-devices/coronavirus-disease-2019-covid-19-emergency-use-authorizations-medical-devices/in-vitro-diagnostics-euas-serology-and-other-adaptive-immune-response-tests-sars-cov-2.

We obtained plasma or serum specimens from CCP donors from March–November 2020. Specimens were shipped, stored, and distributed frozen. All blood donors consented to use of deidentified, residual specimens for further research purposes. Consistent with the policies and guidance of the University of California–San Francisco Institutional Review Board, Vitalant Research Institute self-certified that use of the deidentified CCP donations in this study does not meet the criteria for human subjects research. Centers for Disease Control and Prevention (CDC) investigators reviewed and relied on this determination as consistent with applicable federal law and CDC policy (45 C.F.R. part 46, 21 C.F.R. part 56; 42 U.S.C. § 241[d]; 5 U.S.C. § 552a; 44 U.S.C. § 3501). Qualification for CCP donation required documentation of positive SARS-CoV-2 molecular (e.g., reverse-transcription PCR) or serologic test, complete resolution of symptoms 14–28 days before donation (*9*), and standard allogeneic blood donor qualification criteria (*10*). Samples were selected from CCP donors independent of reactivity on the primary blood donor SARS-CoV-2 screening Ortho VITROS SARS-CoV-2 S Total Ig (Ortho Clinical Diagnostics, https://www.orthoclinicaldiagnostics.com) assay.

To evaluate the waning of reactivity over time, we included longitudinal specimens from 24 CCP donors who continued to qualify for CCP donation at each of 4–14 donations (median 9 donation) over 79–126 days (median 95 days). A COVID-19 seroconversion panel consisted of 14 timepoints from a single-source plasma donor during the progression of a SARS-CoV-2 infection over 87 days (*11*). Fifteen CCP specimens were represented in 6 blinded replicates to evaluate precision. The dilution panel consisted of six 4-fold serial dilutions of specimens with a range of neat antibody titers (*12*). The panel also included 24 apparent serosilent specimens from donors who initially qualified for CCP donation as having a positive molecular test but without evidence of seroconversion by the Ortho S Total Ig assay (https://www.orthoclinicaldiagnostics.com).

### Statistical Analysis

We performed all statistical analyses by using R 4.0.4 (*13*). We used various packages, including the binom package for 95% CIs on proportions (*14*), the glm2 package (*15*) for regression analysis, and the ggplot2 package (*16*) for plotting.

### Sensitivity and Specificity

We assessed sensitivity in cross-sectional CCP specimens. Because data on symptoms, clinical severity, hospitalization, and diagnostic test results (molecular or antigen) were not available, we defined true positivity according to 3 sets of criteria: qualification as a CCP donor according to blood center policies, which required donors to provide evidence of a SARS-CoV-2 diagnosis, with resolved symptomatic infection (n = 191) (https://www.fda.gov/media/141477/download); confirmation of detectable neutralizing antibody by the Broad Institute plaque reduction neutralization test (PRNT) (*12*) (n = 154); or reactive on >3 evaluated binding antibody (bAb) tests (n = 188). Substantial overlap exists between the 3 definitions; 149 specimens were classified as positive by all 3 definitions, 34 by the first and third definitions, 3 by the second and third definitions, and only 12 by only 1 of the definitions. We excluded the 24 serosilent CCP specimens (Table 2) from the sensitivity analysis on the basis of the first criterion. We excluded longitudinal CCP donor cohort specimens from all sensitivity analyses as their continued CCP qualification may bias sensitivity estimates given they were required to have bAb reactivity for continued donation of CCP.

**Table 2:** Composition of the assessment panel for evaluation of SARS-CoV-2 serologic assays in study of commercially available high-throughput SARS-CoV-2 assays for serosurveillance*

Group	Description	No. specimens
Sensitivity subpanels		
Qualification as CCP	191 CCP	191
Broad neutralization activity	152 CCP + 2 serosilent	154
Reactive on >3 assays	186 CCP + 2 serosilent	188
Specificity subpanel	Prepandemic blood donor specimens collected before 2020 and demonstrated to be anti–SARS-CoV-2 negative by RVP neutralization testing	459
Ab persistence subpanel	Longitudinal specimens from 24 donors with at *>*4 CCP donations 84–150 d after index donation	209
Seroconversion subpanel	Longitudinal specimens from a single-source plasma donor with acute SARS-CoV-2 infection	14
Dilutional performance subpanel	Serial dilutions of 5 specimens from sensitivity subpanel; neat (6 replicates), 1:40, 1:80, 1:160, 1:320, and 1:640 analogous to neutralizing antibody testing	55
Serosilent cases	Individual CCP donors nonreactive by S and N anti–SARS-CoV-2 total Ig	24
Repeatability subpanel	Six blinded replicates each of 15 CCP specimens	90

*CCP, coronavirus disease convalescent plasma; N, nucleocapsid; RBD, receptor binding domain; RVP, reporter viral particle; S, spike protein; SARS-CoV-2, severe acute respiratory syndrome coronavirus 2.

We assessed primary specificity with prepandemic blood donor specimens (n = 432) and included 27 seronegative donations from early 2020 (*12*) in a secondary specificity analysis (n = 459) (Appendix Figure 1). Sensitivity and specificity estimates were based on reported qualitative interpretations of assay results. We excluded results reported as equivocal from primary sensitivity and specificity estimates, and conducted secondary analysis that included equivocal results as nonreactive (Appendix Figures 2, 3). All 95% CIs reported are Wilson score intervals.

### Repeatability and Assay Precision

We computed coefficients of variation (CVs), defined as the ratio of the SD to the mean across 6 replicate specimen measurements expressed as a percentage, for each of the replicate specimens (n = 90). A limitation of this approach is that assays with narrower dynamic range produced very low or zero CVs at the upper limit of quantification. To adequately account for reactivity outside the measurement range, these results were excluded from the overall repeatability assessment, and intraclass correlation coefficients (ICCs) were used. The ICC expresses between-sample variance as a proportion of total variance in the tested replicate specimen. In the case of the Bio-Rad BioPlex assay (Bio-Rad, https://www.bio-rad.com), on-board dilutions were conducted by the testing laboratory and used to estimate reactivity in specimens with initial results above the assay’s limit of quantitation.

### Dilutional Performance

The dilution panel (n = 55 specimens) enables comparative assessment of the linearity of observed versus expected reactivity measurements above and below assay cutoffs. Expected reactivity is defined as the mean signal intensity measured over 6 replicates of the neat specimen divided by the dilution factor (Appendix).

### Durability of Antibody Detection

We assessed qualitative and quantitative durability of bAb detection in longitudinal CCP specimens (n = 209 specimens from 24 donors). Documented dates of symptom onset, symptom resolution, or nucleic acid test–based diagnosis are not available for these donors, so all analyses are anchored to the index donation. These CCP donors first donated early in the pandemic, typically within 1 month of symptom resolution (*12*).

We assessed qualitative detection by estimating the proportion of specimens with detectable bAbs grouped in 30-day bins of time since index donations. To account for within-donor correlation, if a donor contributed >1 specimen in a particular time bin, the proportion of the donor’s specimens that were reactive was added to the numerator for the bin and only 1 to the denominator, so that the proportion detected is the proportion of donors detected in each bin.

We assessed quantitative detection by fitting linear mixed effects regression models with time since index donation as the predictor. We estimated assay signal half-lives by transforming average (fixed) slopes obtained from these models (Appendix).

## Results

**Figure 1:**
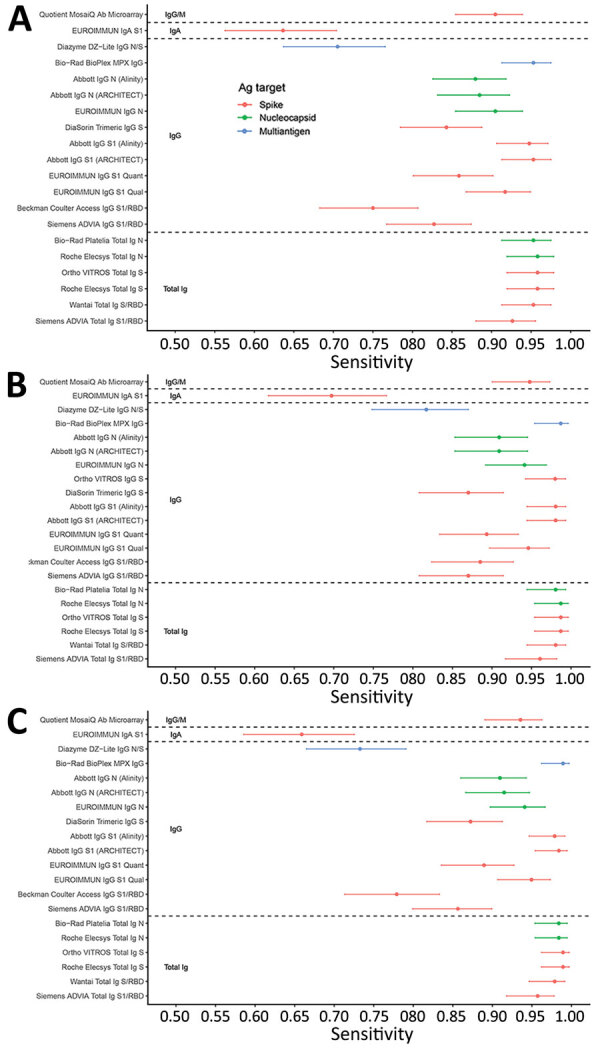
Sensitivity of severe acute respiratory syndrome coronavirus 2 serologic assays (descriptions in Table 1) using 3 definitions of a true positive in study of commercially available high-throughput assays for serosurveillance. A) Positivity defined by qualification as CCP, coronavirus disease convalescent plasma donor (excluding purposely selected serosilent specimens). B) Positivity defined by neutralizing activity measured by Broad plaque reduction neutralization test. C) Positivity defined by operational standard (>3 binding antibody assays reactive). Dots indicate point estimates, and bars indicate Wilson score 95% CIs. Ortho VITROS IgG S assay is included only in panel B because the assay required use of serum for testing; thus, only specimens with available serum and neutralizing data were tested. Ab, antibody; Ag, antigen; N, nucleocapsid; RBD, receptor binding domain; PRNT, plaque reduction neutralization test; S, spike protein.

When a true positive was defined by qualification as a CCP donor, the lowest assay sensitivity was 63.6% (95% CI 56.3%–70.4%) (EUROIMMUN IgA assay, https://www.euroimmun.com), and the highest was 95.8% (95% CI 92.0%–97.9%) (Ortho VITROS Total Ig S assay and Roche Elecsys Total Ig S and N [https://www.roche.com]) (Figure 1, panel A). When a true positive was defined by PRNT activity, the lowest assay sensitivity was 69.7% (95% CI 61.7%–76.7%) (EUROIMMUN IgA assay), and the highest was 98.7% (95% CI 95.4%–99.6%) (Ortho VITROS Total Ig S, Roche Elecsys Total Ig S and N, and Bio-Rad BioPlex MPX IgG assays) (Figure 1, panel B). Most assays (17/20) had sensitivities >80%, 12/20 had sensitivities >90%, and 7/20 had sensitivities >95%, by the first definition. None reached 96% by CCP qualification criteria or 99% by detectable neutralizing antibody criteria. Assays with the lowest sensitivity were the Beckman Coulter Access SARS-CoV-2 IgG (https://www.beckmancoulter.com), Diazyme DZ-Lite SARS-CoV-2 IgG (https://www.diazyme.com), and EUROIMMUN IgA assays, with estimates <80%. We observed similar patterns to the first and second definitions of true positivity when defined by bAb reactivity on >3 assays (Figure 1, panel C).

**Figure 2:**
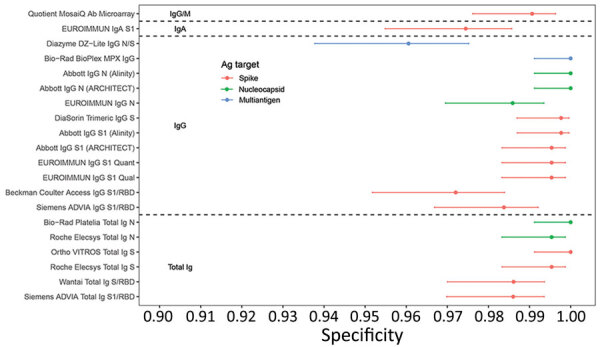
Specificity of severe acute respiratory syndrome coronavirus 2 serologic assays (descriptions in Table 1) in prepandemic negative control specimens in study of commercially available high-throughput assays for serosurveillance. Dots indicate point estimates and bars indicate Wilson score 95% CIs. Ab, antibody; Ag, antigen; N, nucleocapsid; RBD, receptor binding domain; S, spike protein.

Specificities, based on testing 432 prepandemic specimens, were high, with estimates ranging from 96.1% (95% CI 93.8%–7.5%) (Diazyme DZ-Lite assay) to 100% (95% CI 99.1%–100%) (Abbott IgG N [https://www.abbott.com], Bio-Rad BioPlex IgG, Bio-Rad Platelia Total Ig N, and Ortho VITROS Total Ig S assays). Most assays (13/20) had specificities >99%, and 5/20 assays had specificities of 100% in this panel (Figure 2). Assays with poorer specificity tended to have poorer sensitivity, suggesting no tradeoff between sensitivity and specificity (Appendix Table 1, Figure 4). Specificity estimates including 27 specimens from 2020 (Appendix Figure 1) and secondary sensitivity and specificity analyses with equivocal results categorized as nonreactive (Appendix Figures 2, 3) had minimal impact on estimates of sensitivity and specificity.

**Figure 3:**
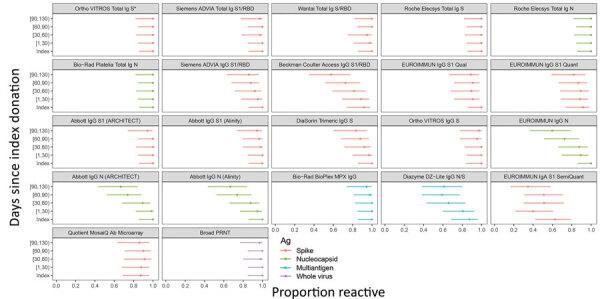
Proportion of donors with detectable severe acute respiratory syndrome coronavirus 2 antibodies in study of commercially available high-throughput assays for serosurveillance. In the longitudinal coronavirus disease convalescent plasma donor cohort, donations were sorted into time bins relative to index donation. Time bin labels on x-axis are denoted with brackets to indicate inclusive boundaries and parentheses to indicate exclusive boundaries. Donors who contributed >1 donation in a time bin contributed the fractional proportion reactive to the numerator and 1 to the denominator for estimation of proportion reactive in the time bin. Symbols indicate point estimates of proportion reactive, and bars indicate 95% CIs (Wilson score). Assays are described in Table 1. Each of the 24 donors had an index sample available. For time bins 1–29 days post index, n = 22 donors, n = 56 specimens; day 30–59, n = 19 donors, n = 45 specimens; day 60–89, n = 22 donors, n = 54 specimens; day 90+, n = 18 donors, n = 30 specimens. Ortho VITROS Total Ig anti-S reactivity was required for qualification of continued CCP donation and therefore shows 100% detection in all time bins by definition. Ab, antibody; Ag, antigen; N, nucleocapsid; PRNT, plaque reduction neutralization test; RBD, receptor binding domain; S, spike protein.

Durability of bAb detection was highly variable, with some assays reactive at all timepoints, whereas others showed substantial declines in the proportion of reactive specimens over time (Figure 3). IgG assays and anti-N assays generally demonstrated more rapid seroreversion proportions compared with total Ig and anti-S assays. For example, the Abbott and EUROIMMUN IgG anti-N assays detected antibodies in <70% of specimens collected >90 days after index donation, whereas total Ig assays like the Ortho Vitros S total Ig and Roche Elecsys N total Ig assays detected antibodies in 100% of specimens at these timepoints. Given the relatively small number of donors in the cohort, the declining detection rates at later timepoints were generally not statistically distinguishable from sensitivity at earlier timepoints for these qualitative assays (χ^2^ tests yielded large p values).

**Figure 4:**
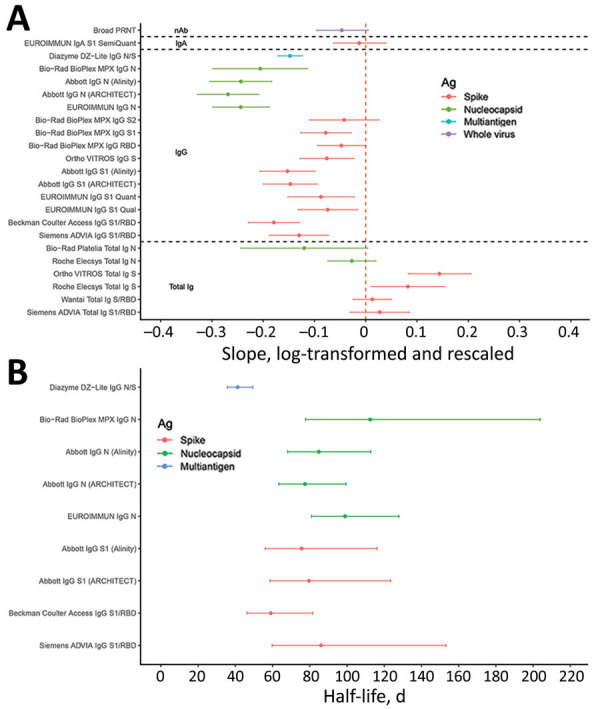
Durability of severe acute respiratory syndrome coronavirus 2 antibody detection as assessed by mixed effects regression modeling in study of commercially available high-throughput assays for serosurveillance. A) Average (fixed) slopes from linear mixed-effects regression models with donor random effects, fit to rescaled and log-transformed quantitative assay signal. B) Assay signal half-lives after index donation for assays demonstrating rapid waning of seroreactivity over time (upper bound on half-life <220 days), estimated on the basis of linear mixed-effects regression models. Assays are described in Table 1. Ab, antibody; Ag, antigen; N, nucleocapsid; RBD, receptor binding domain; S, spike protein.

Regression models of quantitative signal intensity over time showed statistically significant declining reactivity in some assays. All anti-S total Ig (direct antigen sandwich format) assays showed stable or increasing reactivity, whereas all IgG assays showed declining reactivity over time (Figure 4, panel A). Anti-N assays showed more rapid waning than anti-S assays, with multivariable regression confirming that assay format and antigen target are important predictors of rate of waning. Among assays that showed statistically significant declining reactivity, estimated half-lives varied from 41 to 574 days (median 91 days) (Figure 4, panel B).

**Figure 5:**
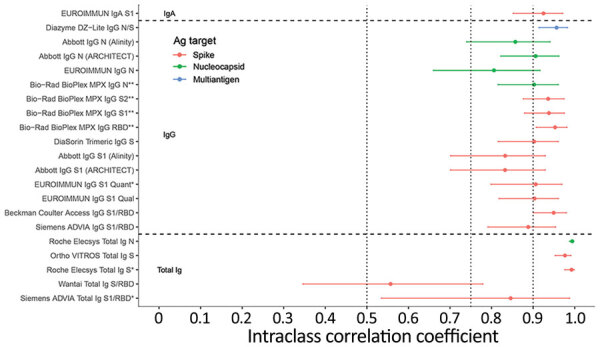
Intraclass correlation coefficients based on blinded replicate sample testing, reflecting the proportion of total variance that is between-sample rather than within-sample variability of severe acute respiratory syndrome coronavirus 2 antibody detection in study of commercially available high-throughput assays for serosurveillance. Results falling outside the primary measurement range were excluded. On-board dilutions were used to estimate reactivity in specimens where initial results fell outside the primary measurement range. Horizontal dotted lines show conventional (although arbitrary) thresholds for moderate (0.5), good (0.75), and excellent (0.9) repeatability (*17*). Assays are described in Table 1. Ab, antibody; Ag, antigen; N, nucleocapsid; PRNT, plaque reduction neutralization test; RBD, receptor binding domain; S, spike protein.

All assays included in the study showed good (ICCs >0.75) or excellent (ICCs >0.9) quantitative repeatability (*17*), with the exception of the Wantai assay (Wantai BioPharm, http://www.ystwt.cn), which had an ICC <0.6 (Figure 5; Appendix Table 2). CVs were generally <10% for low- and medium-titer blinded replicate specimens and somewhat higher for high-titer specimens, ranging from ≈20% to >100% (Appendix Table 3). The Ortho VITROS anti-S and Roche Elecsys anti-N total Ig assays had notably low CVs on most replicate specimens (generally <10%).

Dilutional performance was generally good, with most assays demonstrating reasonable linearity in the relationship between expected and observed reactivity above the assay cutoffs (Appendix Figure 5). Assays with greater dynamic ranges tended to show a linear dilutional response even below the cutoff. Most assays had a well-defined inflection point, representing a level of reactivity below which the dilutional response was not linear.

For most assays all 24 serosilent specimens were nonreactive, whereas 7 assays had 1/24 reactive and 2 assays had 2/24 reactive specimens (Appendix Figure 6). Two of these specimens were reactive on 3 assays, 1 of which was reactive on the 3 EURIMMUN IgG assays; the other had no clear pattern of reactivity (i.e., it was reactive on both IgG and total Ig and S and NC assays). For the single seroconversion series, most assays show seroconversion over the same 2-week timeframe, providing little evidence of variable sensitivity relative to time of infection (Appendix Figure 7). Reporter operator characteristic curve analysis indicated optimal thresholds and corresponding positive and negative percentage agreement for predicting neutralization titers of 1:20, 1:250, and 1:1,000 (Appendix Table 4).

## Discussion

Comprehensive comparison of serologic assays with a broad range of formats, Ig class, and antigen targets are valuable for understanding their relative performance across a range of applications. We focused on application to cross-sectional serosurveillance, although our findings are also informative for other applications. The 3 most critical characteristics for assays used to conduct serosurveillance are sensitivity, including an assay’s ability to detect antibodies after asymptomatic and mildly symptomatic infections, potentially resulting in weak antibody responses (*18*–*20*); specificity, to minimize the effect of false-positives on seroprevalence estimates; and the ability to durably detect antibody responses for accurate estimation of cumulative infections.

For serosurveillance in the context of widespread spike-based vaccine implementation, algorithms that combine S and NC assays with these characteristics can differentiate natural infection from vaccine-induced seroreactivity. In areas with high vaccine-induced anti-S reactivity, single-platform parallel testing is ideal, such as on the Ortho Vitros and Roche Elecsys platforms to maximize throughput and turnaround time. CDC’s Nationwide Blood Donor Seroprevalence Study (https://covid.cdc.gov/covid-data-tracker/#nationwide-blood-donor-seroprevalence) initially screened for anti-S reactivity reflexing reactives to anti-NC testing, and subsequently initiated single-platform simultaneous parallel S and NC testing once anti-S reactivity mainly attributable to vaccination reached high levels to reduce cost and increase efficiency and turnaround time. However, testing algorithms should be context dependent; for example, in regions where whole-virus vaccines are used, alternative algorithms should be considered. The assay performance evaluation we describe informs algorithm development and implementation in different contexts.

Although not specifically applicable to serial cross-sectional serosurveys, the ability to detect breakthrough infections of anti-S–based vaccines in longitudinal datasets is important, requiring sensitive and specific assays to detect development of anti-N reactivity. Detection of reinfection by anamnestic Ab boosting requires quantitative assays with wide dynamic ranges, including the ability to extend the dynamic range through dilution, and assays that demonstrate waning reactivity over time and good quantitative repeatability, such as anti-S and anti-NC IgG.

Ideal assays for serosurveillance applications may not be a viable option in all contexts, and other factors such as cost, logistics, and regulatory approval status may influence assay availability and selection, particularly in resource-constrained settings. However, with robustly characterized assay performance, statistical adjustments can be made in estimating of seroprevalence, such as adjustments for the rate of waning reactivity.

Assay manufacturers commonly determine sensitivity on the basis of timing of seroconversion relative to diagnostic testing or clinical disease, but this criterion may not be the most relevant where high rates of mildly symptomatic or asymptomatic infections exist; alternate definitions of true positivity should be considered. Thus, we used multiple definitions to assess sensitivity in practical serosurveillance contexts. Of particular note, including all CCP donors results in lower sensitivity estimates consequent to inclusion of serosilent infection cases, whereas the requirement for neutralization activity excludes those cases resulting in higher sensitivity estimates.

Detecting past infections long after symptom resolution is key to accurately estimating cumulative incidence of infections based on seroreactivity rates; otherwise, complex adjustments for seroreversion may be required (*3*,*4*,*21*; S. Takahashi et al., unpub. data). Rates of waning immunity are difficult to assess using assays with narrow dynamic ranges that constrain detection of declining reactivity and may plateau at the upper limit of quantitation. Diluting specimens, which many platforms can perform automatically, extends dynamic ranges, enabling quantitation of high-titer specimens, as demonstrated by the Bio-Rad BioPlex assay. Although qualitative seroreversion was observed in some assays, including ones with narrow dynamic ranges (Figure 3), further studies over longer timescales are required. Quantitation of very low-level reactivity is possible in assays demonstrating linearity of dilutional performance below the manufacturer-defined thresholds for reactivity, which are generally set to maintain high specificity. Stable detection of neutralizing activity up to 4 months after index donation demonstrates that in the cross-sectional samples used for sensitivity analysis, waning of neutralizing Ab titers was very unlikely by the time samples were collected and would therefore not have biased sensitivity analyses based on neutralizing activity.

Evaluating of apparent serosilent cases with evidence of prior infection lacking detectable antibodies is important to confirm that this phenomenon is not assay-dependent. Although sporadic reactivity occurred in a few specimens from serosilent cases, most tested nonreactive on all samples, corroborating the findings of other studies (*18*,*22*) indicating some infected persons do not develop a detectable systemic humoral immune response after SARS-CoV-2 infection.

We observed that all anti-S total Ig (direct antigen sandwich format) assays showed stable or increasing reactivity presumably because of continued maturation of antibody affinity, avidity, or both, resulting in increasing signal intensity in these assays (*23*–*25*), whereas all but 1 IgG assay showed declining reactivity over time. Anti-N assays showed more rapid waning than anti-S assays, with multivariable regression confirming that assay format and antigen target are important predictors of rate of waning, although assay format (i.e., Ig target) is a stronger predictor of antibody stability than antigen target. IgG assays demonstrating rapid waning are useful for longitudinal assessment of reactivity relative to neutralizing antibodies and for detecting anamnestic boosting of antibodies because of vaccination or reinfection. Among the IgG assays, the anti-NC assays demonstrated more rapid waning than anti-S assays, which is consistent with observed half-lives of these antibody classes (*26*). IgA assays are not suitable for primary serosurveillance screening; however, because IgA has different detection dynamics than total Ig or IgG assays included in the study, they are informative for detecting incident infections early in infection.

The best performing assays for serosurveillance applications in this evaluation were high-throughput total Ig antigen sandwich format assays, because they met the 3 key performance criteria of durable antibody detection, sensitivity, and specificity. The Ortho and Roche total Ig assays that target anti-S and anti-N antibodies performed well and are currently used in largescale serosurveillance studies including CDC’s Nationwide Blood Donor Seroprevalence Study. The Wantai assay has been widely used in serosurveillance globally (*2*,*5*,*27*); although this assay demonstrated lower specificity and reproducibility than the best performing assays, it performs adequately for serosurveillance after accounting for those limitations. Several other assays, including the Abbott IgG anti-N and EUROIMMUN IgG anti-S assays, have been used in largescale serosurveillance, but require adjustments for rapid waning and seroreversion to estimate cumulative incidence or attack rates, especially over longer periods and multiple epidemic waves (S. Takahashi et al., unpub. data). Our study provides critical data that can be applied to adjust for waning in other studies.

Most assays showed strong correlations of signal intensity with neutralizing titers in cross-sectional specimens, although the IgG assays performed notably better than the rest, and among the IgG assays the anti-S assays showed the highest area under the reporter operator characteristic curve. These assays demonstrated high positive percentage agreement and relatively high negative percentage agreement even at 50% inhibitory dose (ID_50_) >1:1,000. On the basis of these cross-sectional samples collected relatively early after infection, assays with stable or increasing antibody detection over time would show poorer correlation with neutralizing antibody titers, which wane at a similar rate to IgG anti-S assays (Figure 4) and may therefore be less appropriate for identifying correlates of protection.

Our study’s first limitation is that asymptomatic cases are underrepresented in the panel because CCP donors had to qualify on the basis of recovery from symptomatic infection, potentially overestimating sensitivity. The assessment of durability of bAb detection is based on CCP donations from donors whose continued qualification required ongoing Ortho VITROS Total Ig anti-S1 reactivity. Although these CCP donors do not have documented dates of nucleic acid test positivity, symptom onset, or resolution, the first donations were generally within 1–2 months of symptom resolution (*12*). To address these limitations, we developed approaches to adequately characterize sensitivity and durability of reactivity. The study was executed at a time when available postvaccine samples were limited and before the emergence of variants of concern and is therefore constrained by the lack of these sample types (*21*,*28*–*31*). However, this evaluation provides an important foundation for understanding assay performance and their application as the pandemic progresses. The number of specimens included in the dilutional series subpanels are not sufficient for robust assessment of endpoint dilutional sensitivity. The single seroconversion series did not allow for robust assessment of time to seroconversion.

In summary, this study provides a standardized, comparative assessment of 21 SARS-CoV-2 antibody assays from major commercial manufacturers and enables identification of optimal assays and testing algorithms for serosurveillance applications in various contexts. These results also provide performance data applicable to other serologic testing use cases relevant to clinicians, public health organizations, laboratorians, and emergency response planners.

Appendix Additional information about evaluation of commercially available high-throughput SARS-CoV-2 serologic assays for serosurveillance and related applications.
