# Supplementary material for: Evaluation of Commercially Available High-Throughput SARS-CoV-2 Serologic Assays for Serosurveillance and Related Applications
Source: Emerg Infect Dis. 2022 Mar;28(3):672–83. doi: 10.3201/eid2803.211885 (PMC8888213; doi:10.3201/eid2803.211885)
Supplement: Appendix — Additional information about evaluation of commercially available high-throughput SARS-CoV-2 serologic assays for serosurveillance and related applications. [file 21-1885-Techapp-s1.pdf]

Article DOI: https://doi.org/10.3201/eid2803.211885

Evaluation of Commercially Available High-Throughput SARS-Cov-2 Serologic Assays for Serosurveillance and Related Applications

Appendix

Assessing Trends in Quantitative bAb Signal Intensity using Longitudinal Specimens from Repeat COVID-19 Convalescent Plasma (CCP) Donors

Quantitative detection of binding antibodies was assessed by fitting linear mixed effects regression models with time since index donation the only predictor. The regression models included donor random effects (i.e., donor-specific slopes and intercepts) as well as fixed slopes and intercepts. Models were fit to log10-transformed and rescaled assay signal to render slope estimates comparable. Assay signal intensity was first log transformed and then rescaled to an arbitrary scale of 0 to 100, with zero the lowest value observed using a given assay, and 100 the highest value observed with that assay. We report the fixed (average) slopes with 95% confidence intervals in Figure 4, panel A. For comparison, the slope in neutralizing Ab (nAb) titer, measured using the Broad institute plaque reduction neutralization test (PRNT) assay (and rescaled in the same way) is shown. For the single multiplexed assay included in the evaluation, each analyte is reported separately.

In assays with negative slopes that could be clearly distinguished from zero, i.e., where statistical evidence exists of waning reactivity, we estimated signal half-lives by fitting mixed effects regression models for log2-transformed assay signal, and used the fixed effect slopes to estimate half-lives using the following formula:

$$t_{1/2} = -\frac{1}{\beta}$$

with $t_{1/2}$ the half-life and β the regression coefficient on days since index donation. We estimated half-lives only for assays where both point estimates and the upper bound of the 95% confidence interval on the slope were negative.

Appendix Table 1. Sensitivity of SARS-CoV-2 serologic assays using three reference definitions, and specificity in pre-COVID-19 negative control specimens.

Assay	Format	Ag target	Sensitivity (definition 1) (95% CI)	Sensitivity (definition 2) (95% CI)	Sensitivity (definition 3) (95% CI)	Specificity (95% CI)
Siemens ADVIA Total Ig S1/RBD	Total Ig	Spike	92.6% (88.0%–95.6%)	96.1% (91.7%–98.2%)	93.9% (89.7%–96.5%)	98.6% (97.0%–99.4%)
Wantai Total Ig S/RBD	Total Ig	Spike	95.3% (91.3%–97.5%)	98.1% (94.4%–99.3%)	96.5% (92.9%–98.3%)	98.6% (97.0%–99.4%)
Roche Elecsys Total Ig S	Total Ig	Spike	95.8% (92.0%–97.9%)	98.7% (95.4%–99.6%)	97.0% (93.5%–98.6%)	99.5% (98.3%–99.9%)
Ortho VITROS Total Ig S	Total Ig	Spike	95.8% (92.0%–97.9%)	98.7% (95.4%–99.6%)	97.0% (93.5%–98.6%)	100.0% (99.1%–100.0%)
Roche Elecsys Total Ig N	Total Ig	Nucleocapsid	95.8% (92.0%–97.9%)	98.7% (95.4%–99.6%)	96.5% (92.9%–98.3%)	99.5% (98.3%–99.9%)
Bio-Rad Platelia Total Ig N	Total Ig	Nucleocapsid	95.3% (91.3%–97.5%)	98.1% (94.4%–99.3%)	97.0% (93.5%–98.6%)	100.0% (99.1%–100.0%)
Siemens ADVIA IgG S1/RBD	IgG	Spike	82.7% (76.7%–87.4%)	87.0% (80.8%–91.4%)	84.8% (79.2%–89.2%)	98.4% (96.7%–99.2%)
Beckman Coulter Access IgG S1/RBD	IgG	Spike	75.0% (68.3%–80.7%)	88.5% (82.4%–92.7%)	77.5% (71.1%–82.8%)	97.2% (95.2%–98.4%)
EUROIMMUN IgG S1 Qual	IgG	Spike	91.7% (86.8%–94.9%)	94.6% (89.7%–97.2%)	94.1% (89.8%–96.7%)	99.5% (98.3%–99.9%)
EUROIMMUN IgG S1 Quant	IgG	Spike	85.9% (80.1%–90.2%)	89.3% (83.4%–93.3%)	88.5% (83.2%–92.3%)	99.5% (98.3%–99.9%)
Abbott IgG S1 (ARCHITECT)	IgG	Spike	95.3% (91.3%–97.5%)	98.1% (94.4%–99.3%)	96.5% (92.9%–98.3%)	99.5% (98.3%–99.9%)
Abbott IgG S1 (Alinity)	IgG	Spike	94.8% (90.6%–97.1%)	98.1% (94.4%–99.3%)	96.0% (92.2%–97.9%)	99.8% (98.7%–100.0%)
DiaSorin Trimeric IgG S	IgG	Spike	84.3% (78.5%–88.8%)	87.0% (80.8%–91.4%)	85.9% (80.3%–90.0%)	99.8% (98.7%–100.0%)
Ortho VITROS IgG S	IgG	Spike	**	98.0% (94.2%–99.3%)	**	**
EUROIMMUN IgG N	IgG	Nucleocapsid	90.5% (85.4%–93.9%)	94.1% (89.2%–96.9%)	93.4% (89.0%–96.1%)	98.6% (97.0%–99.4%)
Abbott IgG N (ARCHITECT)	IgG	Nucleocapsid	88.5% (83.2%–92.3%)	90.9% (85.3%–94.5%)	89.9% (84.9%–93.4%)	100.0% (99.1%–100.0%)
Abbott IgG N (Alinity)	IgG	Nucleocapsid	88.0% (82.6%–91.8%)	90.9% (85.3%–94.5%)	89.4% (84.3%–93.0%)	100.0% (99.1%–100.0%)
Bio-Rad BioPlex MPX IgG	IgG	Multiantigen	95.3% (91.3%–97.5%)	98.7% (95.4%–99.6%)	97.0% (93.5%–98.6%)	100.0% (99.1%–100.0%)
Diazyme DZ-Lite IgG N/S	IgG	Multiantigen	70.5% (63.7%–76.6%)	81.7% (74.8%–87.0%)	72.6% (66.0%–78.3%)	96.1% (93.8%–97.5%)
EUROIMMUN IgA S1	IgA	Spike	63.6% (56.3%–70.4%)	69.7% (61.7%–76.7%)	65.4% (58.2%–71.9%)	97.4% (95.5%–98.6%)
Quotient MosaiQ Ab Microarray	IgG/IgM	Spike	90.5% (85.4%–93.9%)	94.8% (90.0%–97.3%)	91.8% (87.2%–94.9%)	99.1% (97.6%–99.6%)

Appendix Table 2. Intraclass correlation coefficients based on blinded replicate sample testing, reflecting the proportion of total variance that is between-sample rather than within-sample variability

Assay	Format	Ag target	Intraclass correlation coefficient (95% CI)	N	k
Siemens ADVIA Total Ig S1/RBD*	Total Ig	Spike	0.85 (0.53,0.99)	4	4.4
Wantai Total Ig S/RBD	Total Ig	Spike	0.56 (0.35,0.78)	15	6
Roche Elecsys Total Ig S*	Total Ig	Spike	0.99 (0.98,1.00)	6	4.9
Ortho VITROS Total Ig S	Total Ig	Spike	0.98 (0.95,0.99)	15	6
Roche Elecsys Total Ig N	Total Ig	Nucleocapsid	0.99 (0.99,1.00)	15	6
Siemens ADVIA IgG S1/RBD	IgG	Spike	0.89 (0.79,0.95)	15	5.9
Beckman Coulter Access IgG S1/RBD	IgG	Spike	0.95 (0.90,0.98)	15	5.9
EUROIMMUN IgG S1 Qual	IgG	Spike	0.90 (0.82,0.96)	15	6
EUROIMMUN IgG S1 Quant*	IgG	Spike	0.91 (0.80,0.97)	11	4.8
Abbott IgG S1 (ARCHITECT)	IgG	Spike	0.83 (0.70,0.93)	15	6
Abbott IgG S1 (Alinity)	IgG	Spike	0.83 (0.70,0.93)	15	6
DiaSorin Trimeric IgG S	IgG	Spike	0.90 (0.82,0.96)	15	5.9
Bio-Rad BioPlex MPX IgG RBD**	IgG	Spike	0.95 (0.91,0.98)	15	6
Bio-Rad BioPlex MPX IgG S1**	IgG	Spike	0.94 (0.88,0.98)	15	6
Bio-Rad BioPlex MPX IgG S2**	IgG	Spike	0.94 (0.88,0.97)	15	6
Bio-Rad BioPlex MPX IgG N**	IgG	Nucleocapsid	0.90 (0.82,0.96)	15	6
EUROIMMUN IgG N	IgG	Nucleocapsid	0.81 (0.66,0.92)	15	6
Abbott IgG N (ARCHITECT)	IgG	Nucleocapsid	0.91 (0.82, 0.96)	15	6
Abbott IgG N (Alinity)	IgG	Nucleocapsid	0.86 (0.74,0.94)	15	6
Diazyme DZ-Lite IgG N/S	IgG	Multiantigen	0.96 (0.91,0.98)	15	6
EUROIMMUN IgA S1	IgA	Spike	0.92 (0.85,0.97)	14	5.9

Appendix Table 3. Coefficients of variation reflecting within-sample variability from testing of six blinded replicates of fifteen distinct specimens.

	Sample no.														
Blinded replicate sample	*1*	*2*	*3*	*4*	*5*	*6*	*7*	*8*	*9*	*10*	*11*	*12*	*13*	*14*	*15*
EUROIMMUN IgG S1 Qual	29.8	6	90.2	7.2	6.9	2.1	15.8	14.3	9.1	6.7	8.1	8.5	7.6	7.7	9.4
EUROIMMUN IgG S1 Quant	39.7	8.8	140.7	15.5	12.4	6.3	24.3	26.5	20.9	7.9	***	***	***	***	***
EUROIMMUN IgG N SemiQuant	33.1	12.4	24.8	13.6	13.6	17.1	17.5	26.2	33.6	15.2	6.2	23.4	15.8	14.6	22.6
EUROIMMUN IgA S1 SemiQuant	19.3	23.6	53.6	12.6	9.1	6.5	26.9	18	51.8	8.4	8.5	16.5	8.8	*	23.1
Roche Elecsys Total Ig N Qual	84	4.7	2.8	4.1	***	***	4.4	2.6	4.8	10.8	3.5	7.4	7	3.5	4.3
Roche Elecsys Total Ig S Quant	60.6	4.4	14.4	4.7	7.9	***	5.6	***	***	***	***	***	***	***	***
DiaSorin Trimeric IgG S1/S2 Quant	21.1	37.9	18.3	15.4	21.6	18.9	37.2	33.6	15.7	22.5	11.1	20.9	42.2	11	24.2
Siemens ADVIA Total Ig S1/RBD Qual	12.3	25.8	21.4	***	***	***	***	***	***	***	***	***	***	***	***
Siemens ADVIA IgG S1/RBD Quant	24.8	39.8	20.8	17.1	21.5	20.6	39.7	35.4	8.9	17.4	19.4	23	47.9	7.3	29.9
Abbott IgG N Qual (Alinity)	70.7	17.7	6.6	9.1	6.4	8.9	19.7	31.1	35.8	7.5	8.9	13.1	9.4	12.1	13
Abbott IgG S1 Quant (Alinity)	62.5	19.1	28.4	19	13	19.7	40.1	54.7	15.4	20.4	22.1	37.2	16	14.6	51.1
Abbott IgG N Qual (ARCHITECT)	66.5	15.5	6.1	11.6	9.5	5.4	18.9	33.3	7.7	8.3	***	14.6	4.4	3.5	2.3
Abbott IgG S1 Quant (ARCHITECT)	55.4	21.8	22.6	15.7	20.2	12.2	43	53.4	12.7	19.4	22.4	28.1	14.2	16.4	50.6
Bio-Rad BioPlex MPX IgG RBD	21.4	29.3	19	7.2	11.8	6.5	18.6	16.7	15.9	15.9	5.6	17.8	22.2	10.4	18.4
Bio-Rad BioPlex MPX IgG S1	24	34.9	20.9	6.3	10.4	7.3	18.4	20.7	15.9	13.3	5.4	21.8	25.6	10.8	21
Bio-Rad BioPlex MPX IgG S2	21.1	22.3	**	5	12.9	11.9	19	14.8	10.8	13.3	11.7	20.5	14.2	10	19
Bio-Rad BioPlex MPX IgG N	25.7	30.9	22	5.1	12.7	8.6	20.1	21.3	8.1	13	6.2	20.1	16.1	15	23.8
Bio-Rad Platelia Total Ig N SemiQuant	24.4	11.3	6.6	***	***	***	***	***	***	***	***	***	***	***	***
Beckman Coulter Access IgG S1/RBD	9.5	23.3	11.3	19.2	25	17.2	16.9	44.7	12.9	12.6	16.7	17	21.5	9.8	8.4
Diazyme DZ-Lite IgG N/S	18.6	22.4	22.6	22.3	24.3	30.8	27.1	76.5	8.8	17	25.2	33.3	14.4	37.7	14.1
Wantai Total Ig S/RBD	23.5	8.2	24.7	13.2	13	11.7	11.8	10.8	14.2	77.6	39.3	9.3	4.8	7.6	9.5
Ortho VITROS Total Ig S	11	11.3	7.3	7.3	4.4	7.2	14.6	3.3	13.4	6.4	16.1	6.4	4.6	7.4	7.1

S, spike protein; RBD, receptor binding domain; N, nucleocapsid; Ag, antigen; Ab, antibody; Ig, immunoglobulin. See Table 1 for assay details. Assays with limited dynamic range show some specimens "maxing out" at the maximum of the measurement range, resulting in spuriously low coefficients of variation. Samples with reactivity outside the primary measurement range are excluded. On-board dilutions used to extend the dynamic range of the assay.
*Not tested
**Negative
***CVs not reported because some or all replicates had reactivity outside the measurement range

Appendix Table 4. Optimal thresholds and corresponding positive and negative percent agreement (PPA and NPA) for predicting neutralization titers of 1:20, 1:250, 1:1000 identified through reporter operator characteristic (ROC) curve analysis.

	ID50 >1:20				ID50 >1:250				ID50 >1:1,000			
Assay	*AUC*	*Optimal threshold*	*PPA, %*	*NPA, %*	*AUC*	*Optimal threshold*	*PPA, %*	*NPA, %*	*AUC*	*Optimal threshold*	*PPA, %*	*NPA, %*
Siemens ADVIA Total Ig S1/RBD Qual	0.996	0.88	98.2	100.0	0.827	6.55	75.4	85.0	0.684	9.27	85.1	54.2
Wantai Total Ig S/RBD	0.977	1.03	99.4	87.5	0.808	15.30	83.5	75.0	0.616	13.73	95.7	30.3
Roche Elecsys Total Ig S Quant	0.996	6.48	98.2	100.0	0.877	69.07	79.1	90.0	0.760	245.25	72.3	76.5
Ortho VITROS Total Ig S	0.996	12.61	98.2	100.0	0.847	140.99	79.1	82.5	0.729	279.10	76.6	62.9
Roche Elecsys Total Ig NC Qual	0.990	21.14	92.4	100.0	0.814	82.63	73.4	82.5	0.696	122.40	70.2	68.2
Bio-Rad Platelia Total Ig NC SemiQuant	0.930	1.24	98.8	87.5	0.635	2.58	97.8	32.5	0.565	3.16	100.0	15.9
Siemens ADVIA IgG S1/RBD Quant	0.988	0.84	91.8	100.0	0.862	2.12	85.5	72.5	0.779	10.42	63.8	78.6
Beckman Coulter Access IgG S1/RBD	0.983	0.89	89.9	100.0	0.894	2.88	81.6	82.5	0.843	7.39	85.1	70.5
EUROIMMUN IgG S1 Qual	0.977	1.38	90.6	100.0	0.904	2.82	82.0	85.0	0.829	5.73	72.3	83.3
EUROIMMUN IgG S1 Quant	0.950	8.29	91.2	87.5	0.905	27.21	79.9	87.5	0.841	74.44	76.6	80.3
Ortho VITROS IgG S	0.992	2.27	93.9	100.0	0.896	8.62	78.2	89.5	0.797	11.25	86.7	65.9
Abbott IgG S1 Quant (ARCHITECT)	0.990	149.50	93.0	100.0	0.888	450.50	88.5	75.0	0.819	2821.15	61.7	89.4
Abbott IgG S1 Quant (Alinity)	0.990	112.40	93.0	100.0	0.893	362.15	89.9	75.0	0.827	1586.95	74.5	77.3
DiaSorin Trimeric IgG S Quant	0.986	13.30	89.5	100.0	0.876	44.35	74.1	87.5	0.768	116.00	63.8	80.3

	ID50 >1:20				ID50 >1:250				ID50 >1:1,000			
Assay	*AUC*	*Optimal threshold*	*PPA, %*	*NPA, %*	*AUC*	*Optimal threshold*	*PPA, %*	*NPA, %*	*AUC*	*Optimal threshold*	*PPA, %*	*NPA, %*
EUROIMMUN IgG NC SemiQuant	0.984	0.27	100.0	87.5	0.852	2.25	84.9	77.5	0.741	3.61	80.9	62.1
Bio-Rad BioPlex MPX IgG RBD	0.998	11.00	98.8	100.0	0.886	65.50	87.1	72.5	0.816	198.00	85.1	69.7
Bio-Rad BioPlex MPX IgG S1	0.998	6.00	98.2	100.0	0.907	83.00	69.8	95.0	0.819	155.50	70.2	83.3
Bio-Rad BioPlex MPX IgG S2	0.946	0.75	99.4	87.5	0.866	5.50	72.7	85.0	0.772	5.50	93.6	52.3
Abbott IgG NC Qual (ARCHITECT)	0.985	2.29	87.7	100.0	0.833	3.24	88.5	72.5	0.747	5.79	70.2	68.2
Abbott IgG NC Qual (Alinity)	0.985	2.05	88.3	100.0	0.822	2.77	88.5	72.5	0.731	4.28	83.0	59.8
Bio-Rad BioPlex MPX IgG NC	0.961	4.00	100.0	87.5	0.853	75.50	76.3	85.0	0.805	105.00	85.1	69.7
Diazyme DZ-Lite IgG NC/S	0.899	0.17	97.7	75.0	0.790	1.68	82.7	70.0	0.745	2.80	91.5	52.3
EUROIMMUN IgA S1 SemiQuant	0.945	0.44	90.3	87.5	0.828	1.32	66.9	82.5	0.759	1.32	90.7	56.2

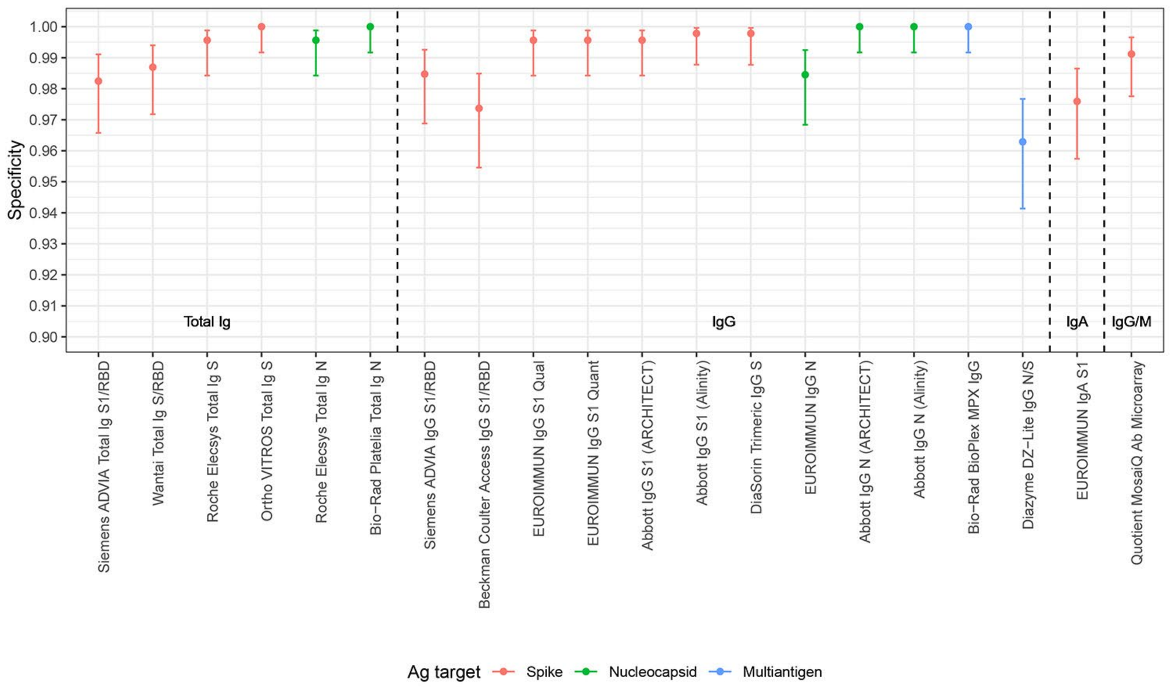

Appendix Figure 1. Specificity of SARS-CoV-2 serologic assays in pre-COVID-19 negative control specimens and early 2020 specimens. S, spike protein; RBD, receptor binding domain; N, nucleocapsid; Ag, antigen; Ab, antibody; Ig, immunoglobulin. See Table 1 for assay details. Dots indicate point estimates and bars indicate Wilson score 95% confidence intervals.

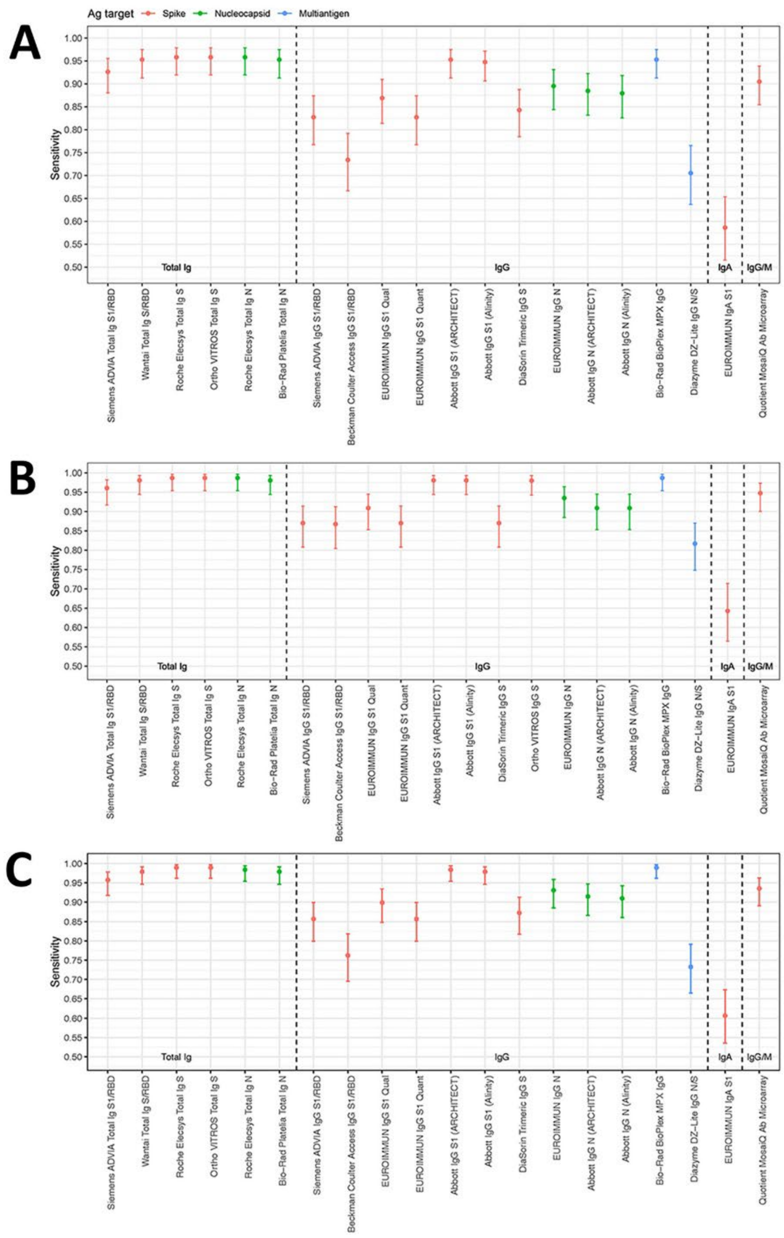

Appendix Figure 2. Sensitivity of SARS-CoV-2 serologic assays using three reference definitions. Secondary analysis with ‘equivocal’ results counted as negative. The following assays had equivocal classifications: EUROIMMUN IgG S1 Qual (*1*), EUROIMMUN IgG S1 Quant (*2*), EUROIMMUN IgG N SemiQuant (*3*), EUROIMMUN IgA S1 SemiQuant (*4*). Panel A: Positivity defined by qualification as CCP donor (excluding purposely selected serosilents). Panel B: Positivity defined by neutralizing activity measured by Broad PRNT. Panel C: Positivity defined by ‘operational standard’ (3 or more bAb assays reactive). Dots indicate point estimates and bars indicate Wilson score 95% confidence intervals. The Ortho VITROS IgG S assay is included only in panel B because it was tested on the subset of specimens with neutralization data available. S, spike protein; RBD, receptor binding domain; N, nucleocapsid; Ag, antigen; Ab, antibody; Ig, immunoglobulin. See Table 1 for assay details.

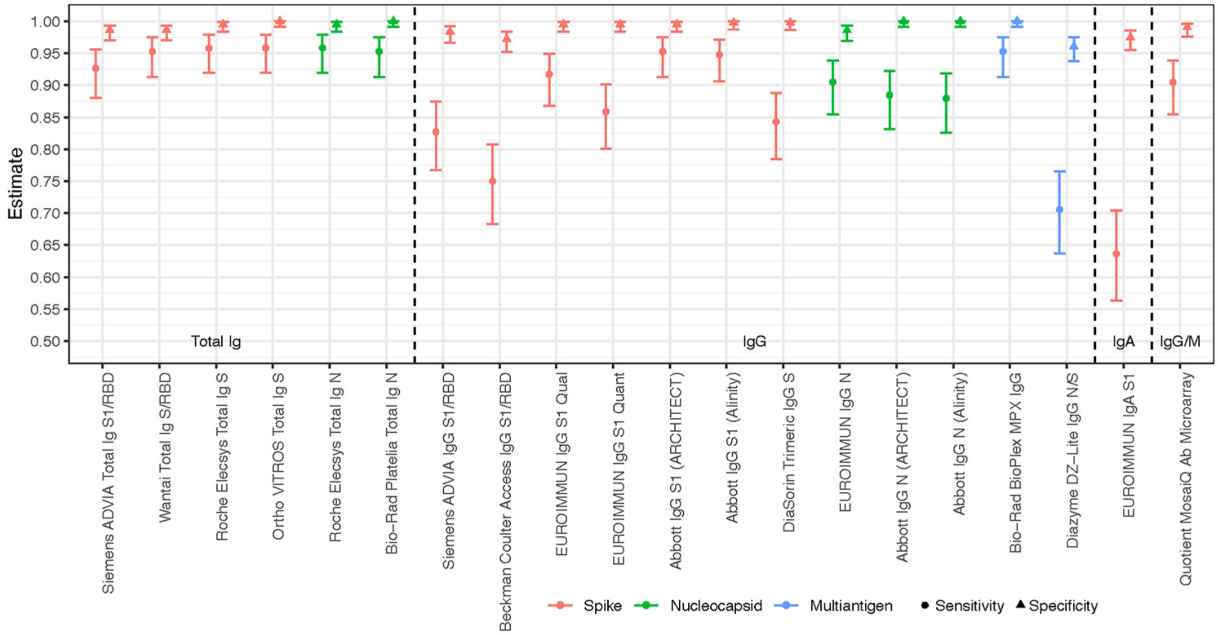

Appendix Figure 3. Specificity of SARS-CoV-2 serologic assays in pre-COVID-19 negative control specimens. Secondary analysis with 'equivocal' and 'borderline' results counted as negative.* S, spike protein; RBD, receptor binding domain; N, nucleocapsid; Ag, antigen; Ab, antibody; Ig, immunoglobulin. The following assays had equivocal classifications: EUROIMMUN IgG S1 Qual (*1*), EUROIMMUN IgG S1 Quant (*2*), EUROIMMUN IgG N SemiQuant (*3*), EUROIMMUN IgA S1 SemiQuant (*4*). * Some assays' instructions for use define a range of signal intensities for which the interpretation is 'equivocal', with intensities above the upper limit of this range interpreted as 'reactive', and those below the lower limit as 'non-reactive'.

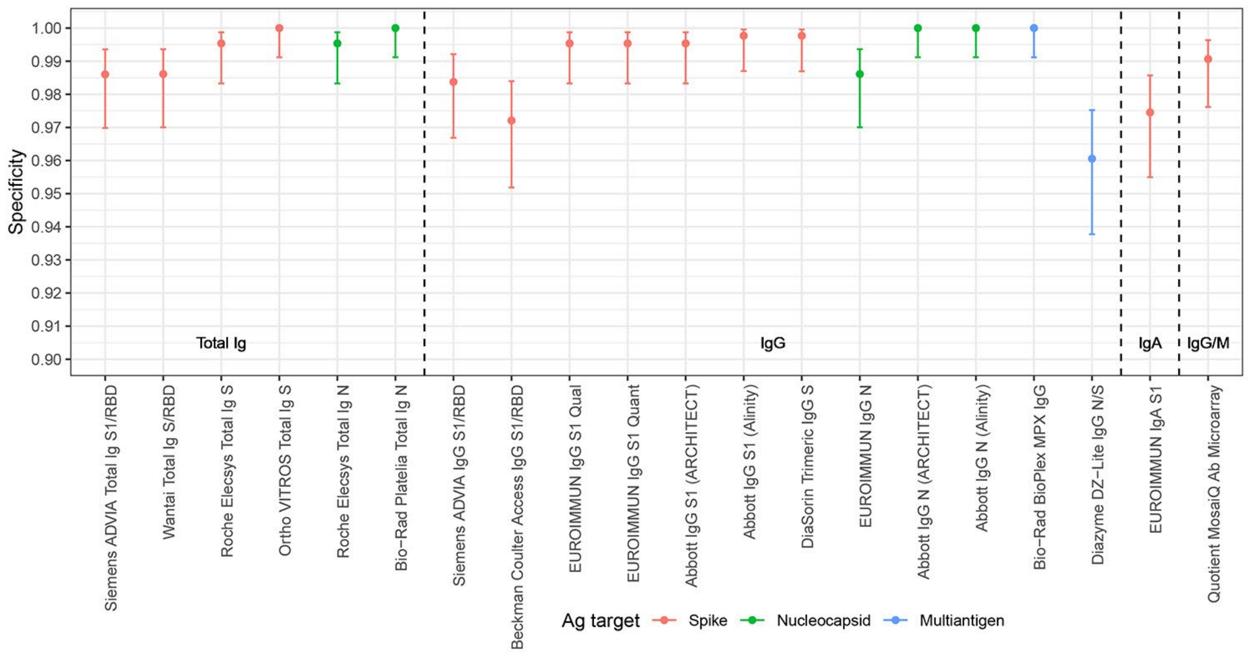

Appendix Figure 4. Sensitivity and specificity of SARS-CoV-2 serologic assays with true positivity defined by reactivity on three or more bAb assays, and Specificity in 2019 pre-COVID-19 specimens. Secondary analysis with ‘equivocal’ and ‘borderline’ results counted as negative.* S, spike protein; RBD, receptor binding domain; N, nucleocapsid; Ag, antigen; Ab, antibody; Ig, immunoglobulin. * Some assays’ instructions for use define a range of signal intensities for which the interpretation is ‘equivocal’, with intensities above the upper limit of this range interpreted as ‘reactive’, and those below the lower limit as ‘non-reactive’.

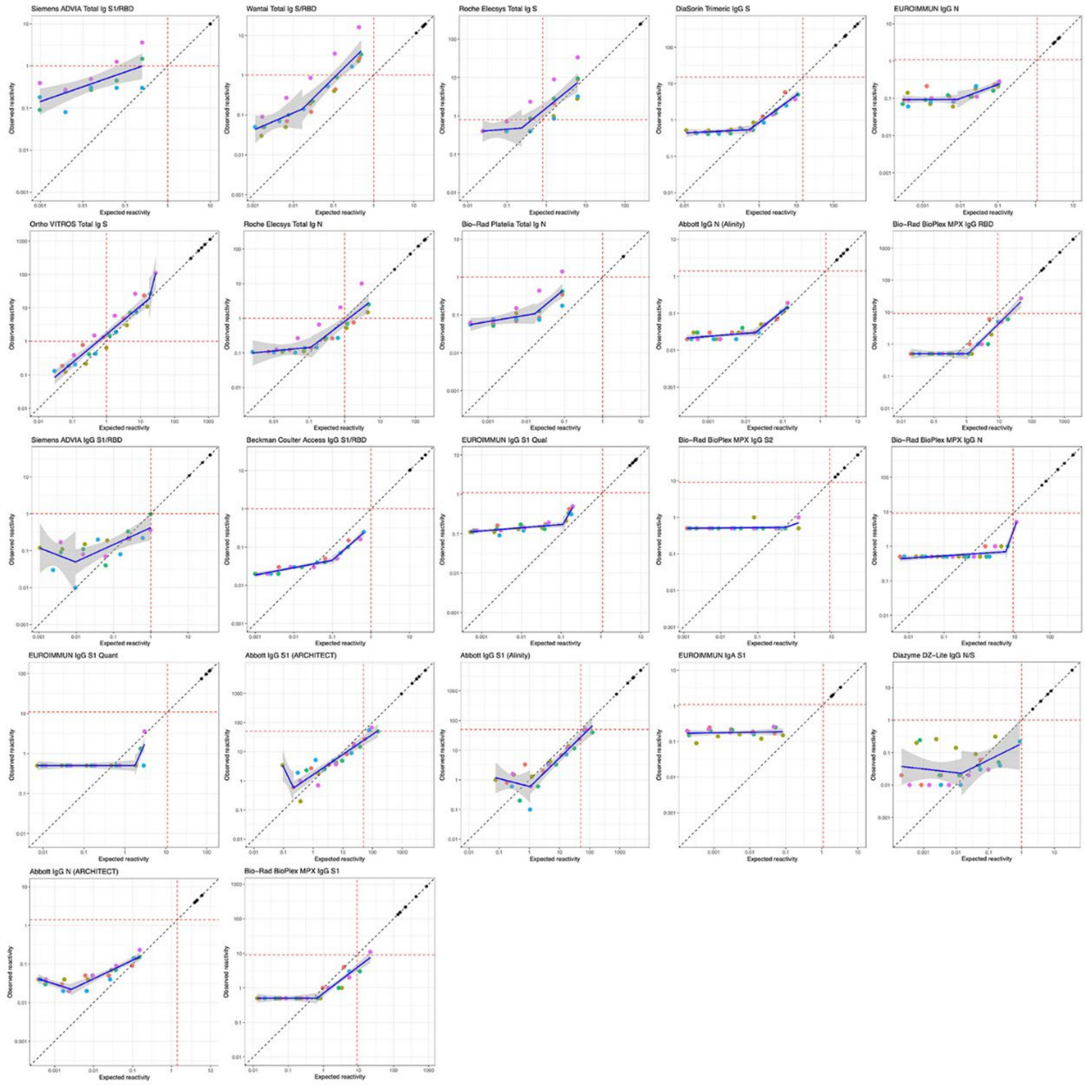

Appendix Figure 5. Observed against expected signal intensity in five 4-fold serial dilutions of specimens with a range of neat Ab titers. S, spike protein; RBD, receptor binding domain; N, nucleocapsid; Ag, antigen; Ab, antibody; Ig, immunoglobulin. Expected reactivity is defined as the mean signal intensity measured over six replicates of the neat specimen divided by the dilution factor.

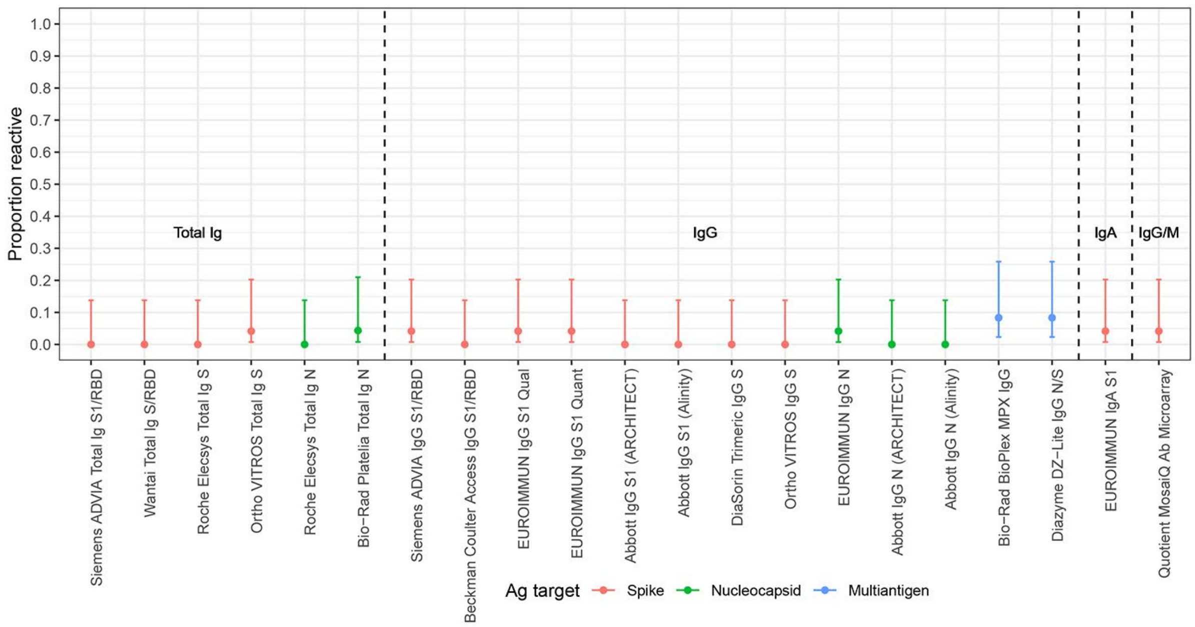

Appendix Figure 6. Proportion of “serosilent” CCP donors (non-reactive on Ortho VITROS Total Ig S during primary blood donor screening) detected by SARS-CoV-2 serologic assays. S, spike protein; RBD, receptor binding domain; N, nucleocapsid; Ag, antigen; Ab, antibody; Ig, immunoglobulin.

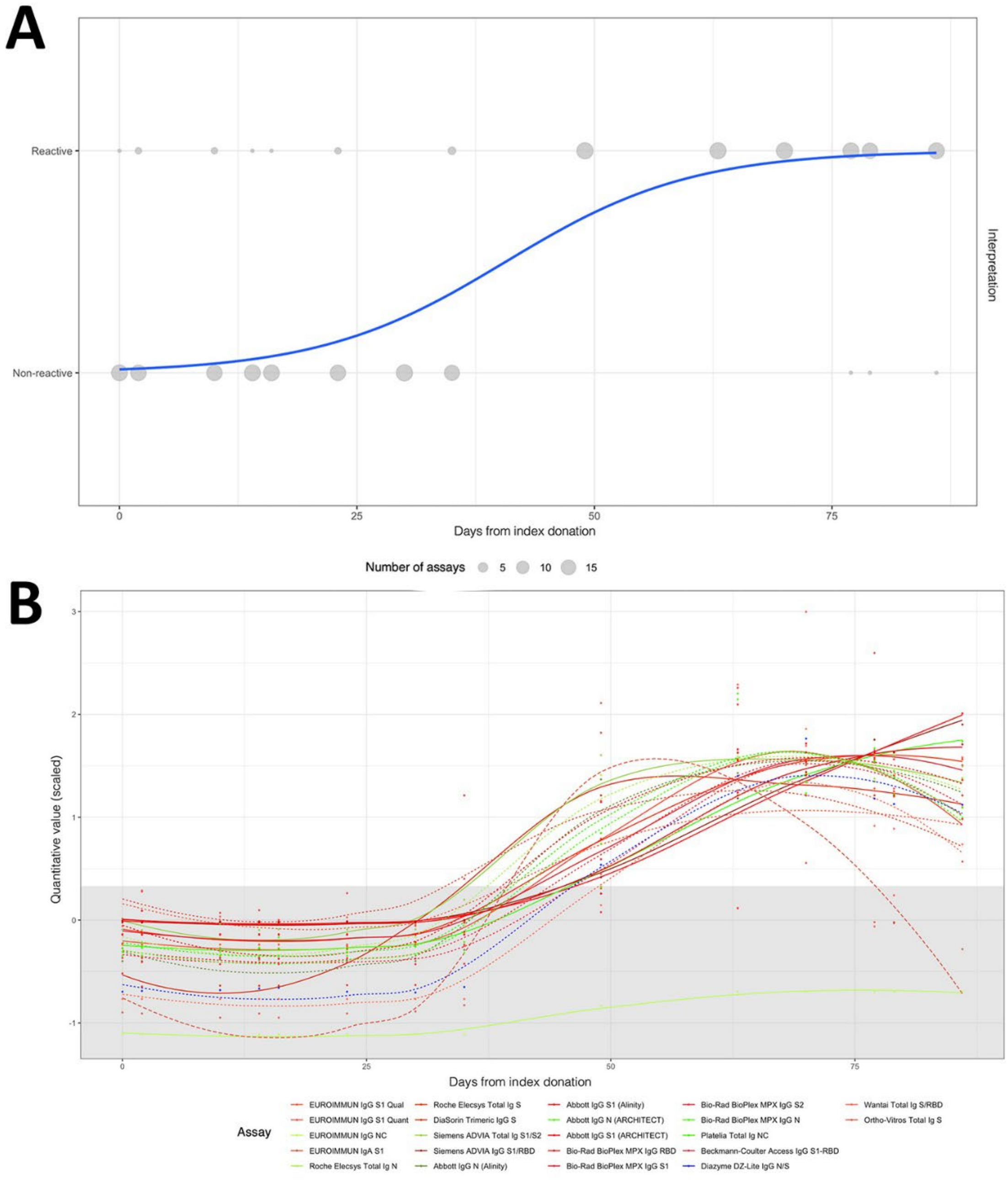

Appendix Figure 7. Relative timing of seroconversion from one seroconversion series without documented date of RT-PCR-based diagnosis. (A) Timing of reactivity by qualitative interpretation. The blue line indicates probability of an assay being reactive at the time point based on logistic regression. (B) Quantitative signal over time. Signal was rescaled and centered to each assay's reactive/non-reactive cutoff and is on an arbitrary scale (−1,3). Shades of red, green, and blue represent spike, nucleocapsid, and spike-nucleocapsid target; respectively. Solid, dot-dashed, and long-dash lines represent total Ig, IgG, and IgA format; respectively. S, spike protein; RBD, receptor binding domain; N, nucleocapsid; Ag, antigen; Ab, antibody; Ig, immunoglobulin.

References

1. US Food and Drug Administration. Investigational COVID-19 convalescent plasma: guidance for industry [cited 2021 Aug 11]. https://www.fda.gov/regulatory-information/search-fda-guidance-documents/investigational-covid-19-convalescent-plasma

2. Hasan T, Pham TN, Nguyen TA, Le HTT, Van Le D, Dang TT, et al. Sero-prevalence of SARS-CoV-2 antibodies in high-risk populations in Vietnam. Int J Environ Res Public Health. 2021;18:6353. PubMed https://doi.org/10.3390/ijerph18126353

3. Pollán M, Pérez-Gómez B, Pastor-Barriuso R, Oteo J, Hernán MA, Pérez-Olmeda M, et al.; ENE-COVID Study Group. Prevalence of SARS-CoV-2 in Spain (ENE-COVID): a nationwide, population-based seroepidemiological study. Lancet. 2020;396:535–44. PubMed https://doi.org/10.1016/S0140-6736(20)31483-5

4. Ibarrondo FJ, Fulcher JA, Goodman-Meza D, Elliott J, Hofmann C, Hausner MA, et al. Rapid decay of anti-SARS-CoV-2 antibodies in persons with mild Covid-19. N Engl J Med. 2020;383:1085–7. PubMed https://doi.org/10.1056/NEJMc2025179
